# Epidemiology, clinical characteristics, and treatment of children with acute intussusception: a case series

**DOI:** 10.1186/s12887-023-03961-y

**Published:** 2023-03-30

**Authors:** Yan Li, Qi Zhou, Chao Liu, Chao Sun, Hao Sun, Xiang Li, Lei Zhang

**Affiliations:** 1 Department of Scientific Research, Cheeloo College of Medicine, Qilu Hospital, Shandong University, Qingdao, China; 2 Department of Pediatric Surgery, Cheeloo College of Medicine, Qilu Hospital, Shandong University, Qingdao, China

**Keywords:** Air enema, Clinical manifestations, Epidemiology, Intussusception, Phloroglucinol

## Abstract

**Background:**

To summarize the clinical and epidemiological characteristics of acute intussusception.

**Methods:**

This retrospective study included pediatric patients with acute intussusception admitted to the Department of Pediatric Surgery, Qilu Hospital (Qingdao), Cheeloo College of Medicine, Shandong University, from January 2014 to December 2019.

**Results:**

A total of 402 infants/children were included (301 males and 101 females) with a mean age of 2.4 ± 1.5 years (2 months to 9 years). Thirty patients (7.5%) had a history of cold food intake, diarrhea, and upper respiratory infection before disease onset. Paroxysmal abdominal pain and crying occurred in 338 patients (84.1%). Eight patients (2.0%) had the typical triad, 167 (41.5%) had vomiting, 24 (6.0%) had bloody stools, and 273 (67.9%) had palpable abdominal mass. The average intussusception depth was 4.0 ± 1.4 cm. Air enema reduction was performed in 344 cases: 335 (97.3%) were successful. Fifty-eight patients were treated with intravenous phloroglucinol (2 mg/kg), and 53 (91.4%) were successful. Sixty-five patients suffered relapses, with a relapse rate of 16.8%.

**Conclusions:**

Pediatric acute intussusception is common. There was no obvious etiology. The clinical manifestations are mostly atypical. Abdominal pain is the most common complaint. Air enema reduction is an effective treatment. The recurrence rate is high.

## Background

Intussusception is an invagination of a proximal segment of the intestine into a distal segment of the intestine that may result in bowel obstruction, venous congestion, and bowel wall edema [1, 2]. It is a common cause of acute abdominal emergencies in infants and children. It is most common in infants and children aged 3 months to 3 years, with a peak incidence between 5 and 9 months of age [1–3]. The average reported annual incidence is 3–40 cases per 10,000 live births in the United States, Europe, and Australia [4–6]. More than 90% of the cases of intussusception in children are idiopathic, but about 5% have a pathological lead point, such as from lymphoid hyperplasia, Meckel diverticulum, duplication cyst, intestinal polyps, mesenteric nodes, lymphoma, surgery, or trauma, that initiates the problem [1, 2, 7]. Most cases are ileocolic, but small bowel and colonic intussusceptions can also occur [1, 2].

The clinical features of intussusception may include any one or more among sudden, intermittent abdominal pain (every 10–15 min), palpable abdominal mass, usually in the right upper quadrant, bilious vomiting, the rectal passage of mucus or blood (“currant jelly” stools), and altered mental status including irritability, crying, lethargy, apathy and/or profound listlessness [1, 2, 8]. The presence of normal bowel sounds does not exclude intussusception [8]. Less common clinical features include painless intussusception, hypovolemic shock, visible peristalsis, diarrhea or constipation, tenesmus, fever, obstruction, sepsis, syncope, and transient hypertension [1, 4, 8].

Intussusception should be treated without delay due to the risk of bowel ischemia and perforation [1, 2]. Intravenous hydration should be given, and surgical consultation should be performed before any attempt [1, 2]. Image-guided pneumatic enema is the preferred first treatment in most cases of intussusception. Hydrostatic enema can also be used for both diagnosis and treatment. Delayed repeat enema can be indicated in cases where the patient remains clinically stable, and the initial enema partially reduces the intussusception, or the intussusception is recurrent. Surgical intervention is indicated in certain cases of intussusception, including peritonitis, free air, shock, sepsis, perforation, repeat enema failure, and persistent symptomatic small-bowel obstruction [1, 2]. Small bowel intussusceptions are not common in children and are often associated with a spontaneous reduction [4]. The risk of mortality following successful treatment of intussusception is low [2]. Recurrent intussusception occurs in up to 10% of cases following enema reduction and 1% of cases following surgical reduction. Repeat enemas are reported to be safe and effective for treating recurrent intussusception if the child remains clinically stable [3, 4]. A recurrence is seen within 48 h in 2.5% of the patients after initial successful air enema reduction [9].

According to the authors’ experience, most children with intussusception need air enema reduction, but children are not very cooperative with enema due to fear and pain. Phloroglucinol is a smooth muscle relaxant that can relax intestinal smooth muscle and relieve intestinal spasms caused by intussusception [10]. Relaxation of intestinal smooth muscle can reduce intestinal spasm and the rapid peristalsis of the intestine, which would lead to the aggravation of intussusception, but also give the opportunity for the intestine to unwind itself.

This study aimed to summarize the clinical and epidemiological characteristics of acute intussusception at a single center, including the use of phloroglucinol.

## Methods

### Study design and population

This retrospective study included pediatric patients with acute intussusception admitted to Qilu Hospital (Qingdao), Cheeloo College of Medicine, Shandong University, from January 2014 to December 2019. The study was approved by the Ethics Committee of Qilu Hospital (Qingdao), Cheeloo College of Medicine, Shandong University (KYLL-qdql2020020). The requirement for informed consent was waived by the committee because of the retrospective nature of the study.

All children were diagnosed by abdominal ultrasound, showing a “target ring sign” or a “concentric circle sign” in the transverse section and a “sleeve sign” in the longitudinal section. The inclusion criteria were (1) age 0–10 years, and (2) diagnosed with intussusception. The exclusion criteria were (1) combined with other surgical acute abdominal conditions, (2) history of previous abdominal surgery, or (3) missing data.

## Data collection

The clinical data of all children were retrospectively collected from historical medical recorders, including sex, age, month of onset, disease duration, etiology, clinical symptoms, intussusception depth, treatments, outcomes, and relapses.

### Statistical analysis

SPSS 19.0 (IBM, Armonk, NY, USA) was used for data analysis. Continuous data were expressed as means ± standard deviation and analyzed using Student’s t-test. Categorical data were expressed as n (%) and analyzed using the chi-square test. Two-sided P-values < 0.05 indicated a statistically significant difference.

## Results

Among the 402 children admitted with acute intussusception, 301 were males, and 101 were females. The average age was 2.4 ± 1.5 years, with the youngest being 2 months old and the oldest 9 years old. Fourteen (3.5%) children were 1–6 months old, 49 (12.2%) were 6–12 months old, 120 (29.8%) were 1–2 years old, 74 (18.4%) were 2–3 years old, 63 (15.7%) were 3–4 years old, 44 (10.9%) were 4–5 years old, and 38 (9.5%) were > 5 years old. The disease onset was most frequent in December (15.2%), but it occurred throughout the year without an obvious central tendency. Most patients did not show an obvious etiology for the disease, while 30 patients (7.5%) had a history of cold food intake, diarrhea, and upper respiratory infection before the onset of the disease. The average time from symptom onset to the initial hospital visit was 13.1 ± 11.0 h, with 277 cases (68.9%) < 12 h, 104 (25.9%) between 12 and 24 h, 18 (4.5%) between 24 and 48 h, and three (0.7%) > 48 h. Among the 402 children, 338 (84.1%) had paroxysmal abdominal pain (young infants presented as paroxysmal crying), and eight patients (2.0%) had a typical triad of intussusception (abdominal pain + bloody stools + abdominal mass). Vomiting occurred in 167 cases (41.5%), bloody stool in 24 (6.0%), and a palpable abdominal mass in 273 (67.9%). The average intussusception depth was 4.0 ± 1.4 cm, with the shortest being 1.1 cm, the longest being 9.0 cm, and the median being 3.9 cm (Table 1).

**Table 1:** Characteristics of the patients

Variables	
Sex	
Male	301 (74.9)
Female	101 (25.1)
Age (months)	
1–6	14 (3.5)
6–12	49 (12.2)
12–24	120 (29.9)
24–36	74 (18.4)
36–48	63 (15.7)
48–60	44 (10.9)
>60	38 (9.5)
Month of onset	
January	30 (7.5)
February	27 (6.7)
March	36 (9.0)
April	26 (6.5)
May	41 (10.2)
June	38 (9.5)
July	27 (6.7)
August	29 (7.2)
September	25 (6.2)
October	29 (7.2)
November	33 (8.2)
December	61 (15.2)
Time from symptom onset to the initial hospital visit	
≤12 h	277 (68.9)
12–24 h	104 (25.9)
24–48 h	18 (4.5)
>48 h	3 (0.7)
Etiology	
History of cold food intake	9 (2.2)
Diarrhea	19 (4.7)
Upper respiratory infection	2 (0.5)
N/A	372 (92.5)
Symptoms	
Paroxysmal abdominal pain/crying	338 (84.1)
Vomiting	167 (41.5)
Bloody stool	24 (6.0)
Abdominal mass	273 (67.9)
Triad	8 (2.0)
Intussusception depth (cm)	4.0 ± 1.4
Treatment	
Air enema	344 (85.6)
Phloroglucinol	58 (14.4)

Data are presented as n (%) or mean ± standard deviation

Among the 402 patients, 344 were treated with air enema reduction, and those not relieved by the initial enema were given repeat air enema. Those who still could not be reduced by delayed air enemas were considered as cases of reduction failure. The cases of reduction success were 335, with a success rate of 97.3%. There were nine cases of reduction failure, which were all successfully reduced by conversion to surgical reduction. Among them, one case of ileo-ileo-colic type and eight cases of ileo-colic type were found. No significant abnormalities were found during the intraoperative probing from the ileocecus to the proximal intestinal canal 1.5 cm in length. Fifty-eight patients were treated with intravenous phloroglucinol (2 mg/kg), and an ultrasound was reviewed 2 h later. The reduction was successful in 53 patients, with a success rate of 91.4%. There were five cases of reduction failure, all of which were successfully reduced after converting to air enema reduction (Table 2).

**Table 2:** Treatment Options and Outcomes

Variable	Air enema reduction (n = 344)	Phloroglucinol (n = 58)	P
Reduction success	335 (97.4)	53 (91.4)	0.037
Reduction failure	9 (2.6)	5 (8.6)	

Data are presented as n (%)

The patients with a > 48 h delay between onset and treatment were due to the parents delaying visiting the hospital or due to travel time to reach the hospital. Still, their overall condition was stable, without high abdominal distension, obvious abdominal tenderness, or abdominal muscle tension, which are not absolute contraindications to air enema. Therefore, they were treated as other patients. Of these three patients, one was cured by air enema (1.5 cm), and two were cured by phloroglucinol (3.1 and 3.6 cm).

Among all successfully treated children, 65 cases suffered relapses, with a relapse rate of 16.8%. Among them, 33 relapsed twice, four relapsed three times, and seven relapsed more than four times. In the highest case, the child relapsed ten times within 2 years. Among those who relapsed more than four times, three were treated with fiberoptic colonoscopy, one had polyps in the terminal ileum, and two had no significant abnormalities.

## Discussion

This study suggested that acute intussusception is common in infants and young children but is also not uncommon in older children. There is no obvious predisposing factor before the onset of the disease, and the clinical manifestations are mostly atypical, with paroxysmal abdominal pain/crying being the most common. Air enema reduction is an effective treatment.

Acute intussusception is generally considered to be more common in kids under 2 years old, especially in infants aged 4 to 9 months [1–3]. Still, in this study, the highest incidence was found in children of 1–2 years, with a proportion of 30%, and the children > 3 and > 5 years old accounted for 36.1% and 9.5%, respectively, suggesting that attention should also be paid to the occurrence of intussusception in older children. Yap Shiyi et al [11]. found that approximately 10% of cases occur in children over five years old, 3–4% in children over ten years old, and 1% in infants less than three months old, supporting the present study. Savoie et al [12]. reported that about 10% of the patients were > 3 years old. Justice et al [13]. reported that 20% of the children were > 1 year old, with the highest age being 6 years.

The etiology and pathogenesis of intussusception are still not fully understood. In the present study, 7.5% of the children had clearly identified predisposing factors. Ntoulia et al [14]. found that around 75% of pediatric acute intussusception cases have no clear predisposing factor. The existing studies suggest that approximately 95% of the intussusceptions are primary, and no significant organic factors can be found in the intestinal segment and its adjacent parts where the intussusception occurs [1, 2, 7]. The majority of predisposing factors for secondary intussusception are considered to be a disturbance of the normal rhythm of intestinal peristalsis, and it is currently believed that its pathogenesis might be related to changes in dietary structure, viral infectious factors, enteritis, and anatomical factors of the ileocecal junction [15–17]. A study in Suzhou showed that intussusception also correlates with meteorological factors such as sunshine, precipitation, and monthly average temperature, [18]. but seasonal changes were not observed in the present study. The high rates of intussusception in older children found in this study suggest that these etiologies may exist locally, but the present study was retrospective, and the exact pathogenesis remains unknown.

The typical presentation of intussusception is paroxysmal abdominal pain/crying, abdominal botuliform mass, and bloody stool. However, in this study, only 6% had bloody stools, only 2% had the typical triad of symptoms, and many older children had only abdominal pain without other symptoms or signs. Therefore, paroxysmal abdominal pain/crying should be the main clinical manifestation of acute intussusception and requires vigilance. Kimia et al [19]. argued that bloody stools are of little diagnostic value in acute intussusception. Justice et al [13]. reported that 78% of the children presented with abdominal pain, lethargy, and vomiting. Another study by Kimia et al [20]. reported that the patterns of symptoms vary with age, with the presence of abdominal pain, emesis, bilious emesis, lethargy, rectal bleeding, and irritability being the predictors in children < 24 months old, while male sex was the only predictor in children > 24 months old. Therefore, for children with atypical clinical symptoms but high suspicion of intussusception, auxiliary examinations (such as abdominal ultrasound) can be performed to assist in the diagnosis.

Ultrasound diagnosis is the preferred method for detecting intussusception in many institutions. Ultrasound can provide insight into the site of intussusception and allow observation of blood flow changes, intestinal dilatation, and ascites [21]. It can also be used to observe the effect of reduction in real time. The sensitivity and specificity of ultrasonography by experienced operators approach 100% [22, 23].

Treatment options for intussusception include non-surgical and surgical treatment. The most commonly used non-surgical treatments include fluoroscopy-guided air enema reduction and ultrasound-guided hydraulic enema reduction. Both have their advantages and disadvantages, with hydraulic enema avoiding the exposure of children to radiation and air enema having the advantages of simplicity, rapidity, and high success rate of reduction. A meta-analysis showed that the success rate of air enemas is 83%, which is higher than the success rate of hydraulic enemas (70%) [24]. In this study, an air enema is the first choice for clinically stable children who had no evidence of intestinal perforation and shock. During the treatment, for children whose intussusception head moves but does not completely disappear with the initial enema and whose general conditions are stable, it may be considered to try again after some time (ranging from 30 min to several hours), which is called delayed repeated enema [25]. Some children in whom surgical reduction is considered due to unsuccessful initial attempts of enema can thus avoid surgery, avoid the occurrence of intestinal adhesion after abdominal surgery, and reduce the economic burden of the children’s family. As for children with advanced intussusception, especially those with poor general conditions or failed air enemas, surgery should be performed promptly. Laparoscopic intussusception reduction is chosen as the surgical method, which has the advantages of less trauma and faster recovery compared with open surgery.

Phloroglucinol is a myotropic non-atropine non-papaverine antispasmodic for smooth muscles and selectively relieves smooth muscle spasms without producing a series of anticholinergic-like adverse effects and has no significant effect on normal smooth muscle [10]. Currently, it is mainly used to treat acute spasmodic pain caused by dysfunction of the digestive system and biliary tract in adults, spasmodic pain of the urethra, bladder, and kidney, and gynecological spasmodic pain [26]. The drug has no obvious toxicity, no obvious teratogenicity, or mutagenicity, which can ensure medication safety [10]. Phloroglucinol has a significant relaxing effect on the intestinal canal with active peristalsis or spasm, relieving abdominal pain and other symptoms and calming the children. The application of phloroglucinol in treating children with acute intussusception is simple and feasible, which can reduce children’s resistance to air enema and is also easily accepted by the parents. In this study, phloroglucinol was effective in managing children with acute intussusception with a depth within 3.9 cm. This study preliminarily suggests the feasibility of phloroglucinol for treating acute intussusception. The authors’ plan is to carry out a clinical trial to apply phloroglucinol to all children with intussusception and use air enema as a salvage treatment if phloroglucinol fails. The relaxation of the intestine could also be conducive to improving the efficacy of air enema.

This study showed that about 16.8% of the children suffered relapses, and the age distribution of the children with relapse was approximately the same as the overall age distribution of the children with the disease. Some studies reported that about 10% of children would relapse after successful non-surgical reduction of intussusception, and the risk of relapse is higher in children > 1 year old [27]. but is not related to the duration of the disease and the reduction technique used [28, 29].

This study has some limitations. First, this study is a single-center retrospective study, limiting the generalizability of the results. Second, this study is retrospective, and there may be bias in data collection. In addition, the analyzable data are limited to those found in the charts. Therefore, a multicenter prospective study with a large sample is needed to provide a sufficient clinical basis in the future.

## Conclusions

In conclusion, acute intussusception is a common acute abdominal disease in children, occurring in infants and young children but also in older children. There was no obvious etiology. The clinical manifestations are atypical in most cases. Abdominal pain is the most common complaint from children presenting with intussusception. Air enema reduction is an effective treatment. Phloroglucinol might also be considered. The recurrence rate is high.

## Data Availability

All data generated or analyzed during this study are included in this published article.

## References

[1] Loukas M, Pellerin M, Kimball Z, de la Garza-Jordan J, Tubbs RS, Jordan R (2011). Intussusception: an anatomical perspective with review of the literature. Clin Anat.

[2] Edwards EA, Pigg N, Courtier J, Zapala MA, MacKenzie JD, Phelps AS (2017). Intussusception: past, present and future. Pediatr Radiol.

[3] Applegate KE (2009). Intussusception in children: evidence-based diagnosis and treatment. Pediatr Radiol.

[4] Ko HS, Schenk JP, Troger J, Rohrschneider WK (2007). Current radiological management of intussusception in children. Eur Radiol.

[5] Tate JE, Simonsen L, Viboud C, Steiner C, Patel MM, Curns AT (2008). Trends in intussusception hospitalizations among US infants, 1993–2004: implications for monitoring the safety of the new rotavirus vaccination program. Pediatrics.

[6] Buettcher M, Baer G, Bonhoeffer J, Schaad UB, Heininger U (2007). Three-year surveillance of intussusception in children in Switzerland. Pediatrics.

[7] Yang G, Wang X, Jiang W, Ma J, Zhao J, Liu W (2013). Postoperative intussusceptions in children and infants: a systematic review. Pediatr Surg Int.

[8] Waseem M, Rosenberg HK (2008). Intussusception Pediatr Emerg Care.

[9] Simanovsky N, Issachar O, Koplewitz B, Lev-Cohain N, Rekhtman D, Hiller N (2019). Early recurrence of ileocolic intussusception after successful air enema reduction: incidence and predisposing factors. Emerg Radiol.

[10] Jung H, Kim HJ, Choi ES, Lee JY, Park KS, Cho KB (2021). Effectiveness of oral phloroglucinol as a premedication for unsedated esophagogastroduodenoscopy: a prospective, double-blinded, placebo-controlled, randomized trial. PLoS ONE.

[11] Yap Shiyi E, Ganapathy S (2017). Intussusception in Children presenting to the Emergency Department: an asian perspective. Pediatr Emerg Care.

[12] Savoie KB, Thomas F, Nouer SS, Langham MR, Huang EY (2017). Age at presentation and management of pediatric intussusception: a Pediatric Health Information System database study. Surgery.

[13] Justice FA, Auldist AW, Bines JE (2006). Intussusception: trends in clinical presentation and management. J Gastroenterol Hepatol.

[14] Ntoulia A, Tharakan SJ, Reid JR, Mahboubi S (2016). Failed Intussusception reduction in children: correlation between Radiologic, Surgical, and pathologic findings. AJR Am J Roentgenol.

[15] Jain S, Haydel MJ. Child Intussusception. StatPearls. Treasure Island (FL)2022. 28613732

[16] Kaemmerer E, Tischendorf JJ, Steinau G, Wagner N, Gassler N (2009). Ileocecal intussusception with histomorphological features of inflammatory neuropathy in adenovirus infection. Gastroenterol Res Pract.

[17] Bogdanovic M, Blagojevic M, Kuzmanovic J, Jecmenica D, Alempijevic D (2019). Fatal intussusception in infancy: forensic implications. Forensic Sci Med Pathol.

[18] Guo WL, Zhang SF, Li JE, Wang J, Wang LJPO. Association of Meteorological Factors with Pediatric Intussusception in Subtropical China: A 5-Year Analysis. 2014;9:e90521-. 10.1371/journal.pone.0090521 PMC3938762 24587386

[19] Kimia AA, Williams S, Hadar PN, Landschaft A, Porter J, Bachur RG (2018). Positive guaiac and bloody stool are poor predictors of intussusception. Am J Emerg Med.

[20] Kimia AA, Hadar PN, Williams S, Landschaft A, Monuteaux MC, Bachur RG (2020). Variation in the presentation of Intussusception by Age. Pediatr Emerg Care.

[21] Bartocci M, Fabrizi G, Valente I, Manzoni C, Speca S, Bonomo L (2015). Intussusception in childhood: role of sonography on diagnosis and treatment. J Ultrasound.

[22] Hryhorczuk AL, Strouse PJ (2009). Validation of US as a first-line diagnostic test for assessment of pediatric ileocolic intussusception. Pediatr Radiol.

[23] Charles T, Penninga L, Reurings JC, Berry MC (2015). Intussusception in children: a clinical review. Acta Chir Belg.

[24] Sadigh G, Zou KH, Razavi SA, Khan R, Applegate KE (2015). Meta-analysis of Air Versus Liquid Enema for Intussusception reduction in children. AJR Am J Roentgenol.

[25] Lautz TB, Thurm CW, Rothstein DH (2015). Delayed repeat enemas are safe and cost-effective in the management of pediatric intussusception. J Pediatr Surg.

[26] Chassany O, Bonaz B, Bruley DESVS, Bueno L, Cargill G, Coffin B (2007). Acute exacerbation of pain in irritable bowel syndrome: efficacy of phloroglucinol/trimethylphloroglucinol. A randomized, double-blind, placebo-controlled study. Aliment Pharmacol Ther.

[27] Yu Y, Qin X, Yan S, Wang W, Sun Y, Zhang M (2015). Non-leukemic myeloid sarcoma involving the vulva, vagina, and cervix: a case report and literature review. Onco Targets Ther.

[28] Kim JH, Lee JS, Ryu JM, Lim KS, Kim WY (2018). Risk factors for recurrent intussusception after fluoroscopy-guided Air Enema. Pediatr Emerg Care.

[29] Justice FA, Nguyen LT, Tran SN, Kirkwood CD, Thi NT, Carlin JB (2011). Recurrent intussusception in infants. J Paediatr Child Health.

